# Abdominal intra-compartment syndrome – a non-hydraulic model of abdominal compartment syndrome due to post-hepatectomy hemorrhage in a man with a localized frozen abdomen due to extensive adhesions: a case report

**DOI:** 10.1186/s13256-016-1045-x

**Published:** 2016-09-15

**Authors:** Alexsander K. Bressan, Andrew W. Kirkpatrick, Chad G. Ball

**Affiliations:** 1 Department of Surgery, University of Calgary and the Foothills Medical Centre, North Tower 10th Floor, 1403-29th Street Northwest, Calgary, AB T2N 2T9 Canada; 2 Departments of Surgery and Oncology, Foothills Medical Centre and the University of Calgary, 1403 – 29 Street NW, Calgary, AB T2N 2T9 Canada

**Keywords:** Abdominal compartment syndrome, Abdominal intra-compartment syndrome, Intra-abdominal pressure, Intra-abdominal hypertension, Abdominal compliance, Hydraulic model, Case report

## Abstract

**Background:**

Postoperative hemorrhage is a significant cause of morbidity and mortality following liver resection. It typically presents early within the postoperative period, and conservative management is possible in the majority of cases. We present a case of late post-hepatectomy hemorrhage associated with overt abdominal compartment syndrome resulting from a localized functional compartment within the abdomen.

**Case presentation:**

A 68-year-old white man was readmitted with sudden onset of upper abdominal pain, vomiting, and hemodynamic instability 8 days after an uneventful hepatic resection for metachronous colon cancer metastasis. A frozen abdomen with adhesions due to complicated previous abdominal surgeries was encountered at the first intervention, but the surgery itself and initial recovery were otherwise unremarkable. Prompt response to fluid resuscitation at admission was followed by a computed tomography of his abdomen that revealed active arterial hemorrhage in the liver resection site and hemoperitoneum (estimated volume <2 L). Selective arteriography successfully identified and embolized a small bleeding branch of his right hepatic artery. He remained hemodynamically stable, but eventually developed overt abdominal compartment syndrome. Surgical exploration confirmed a small volume of ascites and blood clots (1.2 L) under significant pressure in his supramesocolic region, restricted by his frozen lower abdomen, which we evacuated. Dramatic improvement in his ventilatory pressure was immediate. His abdomen was left open and a negative pressure device was placed for temporary abdominal closure. The fascia was formally closed after 48 hours. He was discharged home at postoperative day 6.

**Conclusions:**

Intra-abdominal pressure and radiologic findings of intra-abdominal hemorrhage should be carefully interpreted in patients with extensive intra-abdominal adhesions. A high index of suspicion and detailed understanding of abdominal compartment mechanics are paramount for the timely diagnosis of abdominal compartment syndrome in these patients. Clinicians should be aware that abnormal anatomy (such as adhesions) coupled with localized pathophysiology (such as hemorrhage) can create a so-named abdominal intra-compartment syndrome requiring extra vigilance to diagnose.

## Background

Abdominal compartment syndrome (ACS) is characterized by progressive organ-system failure due to increased intra-abdominal pressure (IAP). Any cause of intra-abdominal hypertension (IAH) can potentially lead to ACS, although it is most common in both injured and critically ill patients. The reported incidence of ACS is extremely variable (20 to 80 %) due to heterogeneous diagnostic criteria and study populations [1]. For this reason the Abdominal Compartment Society has attempted to standardize a working definition for ACS [2, 3], and to revise the definition as required [4]. Risk factors include, but are not limited to, aggressive fluid resuscitation with crystalloids, polytransfusion, coagulopathy, and sepsis [1, 5, 6].

Following major elective abdominal surgery, IAH has been reported in 12 % of patients [7], most often within 72 hours after the operation [8]. The prognostic importance of IAH alone remains controversial [1, 7, 9, 10], and progression to ACS is typically reported in association with large ventral hernia repair or multiple postoperative complications (prolonged ileus, gastric dilatation, ascites, anastomotic leakages, intra-abdominal infection, hemorrhage).

In hepatobiliary surgery, postoperative hemorrhage is a major cause of surgical morbidity and mortality. The reported incidence varies from 0.6 to 8 % [11, 12]. It typically presents within 48 hours of surgery with blood in a drain, a decrease in hemoglobin levels, and/or hemodynamic instability. Management is most often conservative, although a repeat laparotomy is indicated for massive or refractory bleeding. To the best of our knowledge, no case of ACS due to postoperative hemorrhage has been reported in elective hepatobiliary surgery.

We thus report a case of late post-hepatectomy, small volume hemorrhage associated with ACS within a compartmentalized peritoneal cavity resulting from severe intra-abdominal adhesions, which constitutes abdominal intra-compartment syndrome.

## Case presentation

A 68-year-old white man presented to our Emergency Department with sudden onset of upper abdominal pain and vomiting 8 days after undergoing an elective hepatic resection via a bilateral subcostal incision. He had been discharged home the day before following an uneventful hepatic bisegmentectomy (4 and 5), cholecystectomy, and extensive perihepatic adhesiolysis for a metachronous colon cancer metastasis. His past history was significant for a transverse colectomy with end colostomy and mucous fistula 2 years earlier for an obstructive, moderately differentiated, Stage IIIC (pT3pN2b) adenocarcinoma. His recovery from the colorectal surgery was complicated with surgical site infection and fascia dehiscence requiring three reoperations. Adjuvant leucovorin, fluorouracil, and oxaliplatin (FOLFOX) chemotherapy was completed without significant side effects and the colostomy was reversed.

**Fig. 1:**
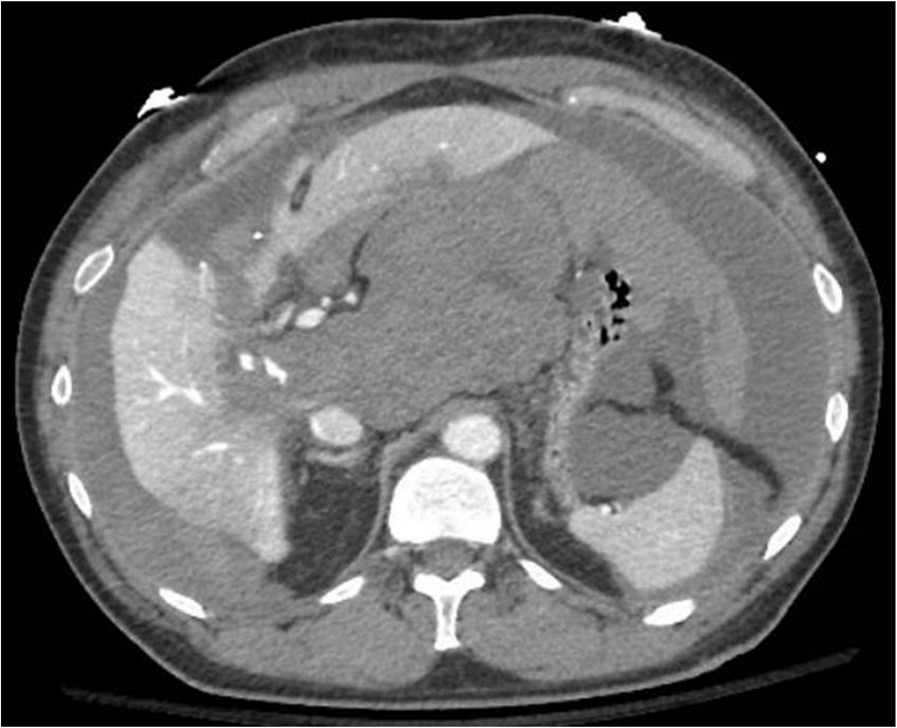
Computed tomography of the abdomen showing heterogeneous fluid (blood and ascites) in the perihepatic and perisplenic regions, and a hematoma in the lesser sac

**Fig. 2:**
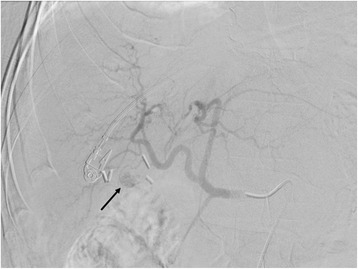
Selective common hepatic arteriogram indicating a small branch of the right hepatic artery supplying the active site of bleeding (*arrow*)

An initial examination revealed that he was alert, body mass index (BMI) 25.6 kg/m^2^, mildly pale, afebrile, and dyspneic, with hemodynamic instability (blood pressure 90/60 mmHg, heart rate 112). His recent bilateral subcostal incision had been closed with skin staples and was healing without complications. Surgical scars from former bilateral stomas and midline laparotomy were normal. His abdomen was tender upon palpation of the right upper quadrant. Laboratory findings included: hemoglobin 8.9 g/dL; lactate 3.3 mmol/L; and creatinine 1.83 mg/dL. Arterial blood gas values showed: pH 7.30, partial pressure of carbon dioxide (pCO_2_) 27 mmHg, partial pressure of oxygen (pO_2_) 95 mmHg, and bicarbonate (HCO_3_) 13 mmol/L (on 3 liters of supplemental oxygen per minute). Hypotension and tachycardia promptly responded to intravenous volume expansion with 2 liters of normal saline and 2 units of packed red blood cells, but no urine output was present. A triphasic computed tomography of his abdomen was obtained, and a small to moderate volume hemoperitoneum (predominantly in the lesser sac and perihepatic regions) was identified (Fig. 1). Celiac arteriography also defined a small bleeding branch of his right hepatic artery (Fig. 2), which was arrested with selective transcatheter arterial embolization. He remained hemodynamically stable after the procedure, and he was admitted to our Surgery Unit.

Four hours following presentation, he complained of mild upper abdominal pain and presented minimal urine output, but he was otherwise comfortable on 3 liters of supplemental oxygen per minute, intravenous crystalloids, and analgesics. Laboratory results showed: hemoglobin 11.2 g/dl, creatinine 1.8 mg/dl, pH 7.30, pCO_2_ 32 mmHg, pO_2_ 81 mmHg, and HCO_3_ 16 mmol/L.

Over the subsequent 24 hours, his hemodynamic and hematologic parameters remained stable, but his urine output was still minimal, and he developed worsening upper abdominal pain and respiratory failure. A physical examination revealed a tense abdomen with signs of associated ventilatory restriction. Laboratory results showed: pH 7.43, pCO_2_ 34 mmHg, pO_2_ 80 mmHg, HCO_3_ 23 mmol/L (on 15 liters per minute of supplemental oxygen on high flow mask), lactate 2.4 mmol/L, and creatinine 4.86 mg/dL. In this setting of abdominal distension with evolving restrictive respiratory failure, and acute kidney injury, an IAP measurement of 21 mmHg was obtained via a three-way urinary catheter, by the end of expiration, and in the absence of abdominal contractions. Sustained IAH was confirmed by repeated IAP measurements and a diagnosis of ACS was made, approximately 28 hours from readmission. He was promptly transferred to our Intensive Care Unit to receive sedation and ventilatory support; follow-up IAP measures at 21 mmHg were again obtained. The mechanics of IAH were felt to be poorly explained by such a limited volume of intra-abdominal fluid (estimated on computed tomography to be far less than 2 liters), but considering the imaging findings of a dominant central collection of blood clots in his lesser sac (Fig. 1) and accelerated clinical deterioration, surgical decompression was indicated.

**Fig. 3:**
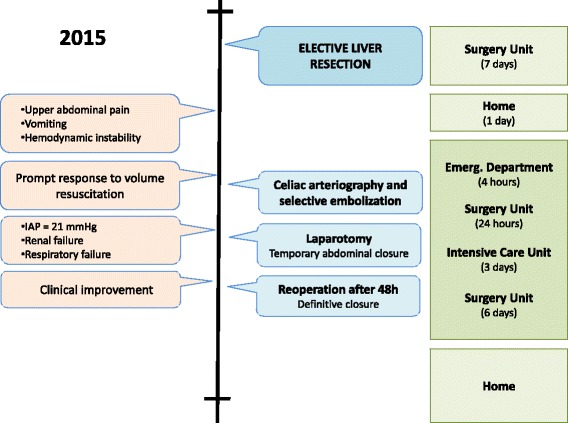
Timeline of interventions and outcomes. *Emerg.* Emergency, *h* hours, *IAP* intra-abdominal pressure

Within 2 hours following the diagnosis of ACS, surgical decompression was performed. A surgical approach through his recent bilateral subcostal incision allowed direct access to his hemoperitoneum, which was confined to the recently dissected perihepatic region. The remaining peritoneal space was completely obliterated with firm adhesions and defined to be surgically inaccessible. A total of 1.2 liters of blood clots and ascites under significant pressure were evacuated. Dramatic improvement in his ventilatory pressure was immediate (peak airway pressures decreased from 37 cmH_2_O to 20 cmH_2_O; tidal volumes increased from 120 ml to 450 ml). The surgical site was irrigated with saline, the fascia was left open, and a negative pressure device was placed for temporary abdominal closure. After the operation he was returned to our Intensive Care Unit, and extubation was possible within 24 hours, in the presence of normal hemodynamics and improving renal function (urine output >1 ml/kg/minute; creatinine 2.86 mg/dl). The fascia was formally closed within 48 hours without complication. Renal replacement therapy was not necessary, and he was discharged home the following week (Fig. 3).

## Discussion

Pascal’s law states that a pressure change anywhere in an enclosed incompressible fluid is transmitted uniformly without loss to every portion of the fluid and container walls. In such a system, pressure should also be equal in all points at the same absolute height. The pressure difference between two points is also defined by the weight of the fluid column between them. Application of this hydraulics principle to the abdominal cavity allows us to conceptualize IAP and standardize its measurement technique.

The former World Society of Abdominal Compartment Syndrome, now known as the Abdominal Compartment Society, defines IAP as the steady-state pressure concealed within the abdominal cavity [4]. Indirect measurement via a urinary catheter is recommended as a safe and reproducible method. Sustained elevated measures >20 mmHg in association with new organ failure define ACS. Added volume of abdominal contents (intraluminal or extraluminal) is the basic underlying mechanism driving IAP elevation, and abdominal compliance is a major parameter that influences volume–pressure association [13].

Abdominal compliance represents the intra-abdominal volume variation in response to a change in IAP; it reflects the elasticity of flexible parts of abdominal cavity boundaries (abdominal wall and diaphragm). Increased abdominal compliance allows greater volume accommodation and a less steep rise in the volume–pressure curve. Decreased elasticity translates into lower compliance, and it is associated with old age; male gender; short stature; central obesity; prone position; and abdominal wall scars, hematomas, and muscle hypertrophy [14].

However, the estimation of volume–pressure effects of acute fluid accumulation in the abdomen remains poorly explored in clinical practice. *In vivo* studies defining the effects of pneumoperitoneum during laparoscopic surgery have been conducted, but factors such as compressibility of CO_2_, patient position, use of neuromuscular blockage, and mechanical ventilation should be considered distinct from a fluid-based scenario. Mulier *et al*. reported that an average of 3 liters (2 to 4.4 L) of CO_2_ was insufflated in the abdominal cavity of 20 patients in straight supine position before reaching a preset pressure limit of 15 mmHg [15]. Also, a steep decrease in abdominal compliance has been demonstrated as the abdominal wall approaches maximal stretching, at around 15 mmHg [16]. Thereafter, initial linear pressure-volume association gives place to an exponential curve, and clinical effects follow an accelerated pace [13].

Association of ACS with aggressive fluid resuscitation has also been described, particularly in patients with secondary ACS (that is, no primary abdominal pathology) [17, 18]. In a prospective cohort of major torso trauma, Balogh and colleagues identified volume of crystalloids to be an independent predictor of poor outcomes in patients with secondary ACS [6]. In another prospective study of a critically ill medical population, 85 % of patients with a net positive fluid balance greater than 5 L in 24 hours developed IAH, and one-third of them were diagnosed with ACS. A statistically significant association of positive fluid balance and ACS in mixed medical and surgical populations was also demonstrated in a prospective cohort by Vidal *et al*. (relative risk = 2.5) [5], and in a systematic review by Holodinsky *et al*. (odds ratio = 5.2) [19].

Goal-directed fluid resuscitation using dynamic parameters (for example, pulse pressure variation, stroke volume variation) has been suggested [20], since the accuracy of traditional pre-load parameters (for example, central venous pressure, pulmonary artery wedge pressure) is blunted by IAH. However, threshold values of functional hemodynamic parameters for fluid responsiveness in IAH still need better characterization, and the real benefit remains uncertain [20, 21]. The use of abdominal perfusion pressure (difference between mean arterial pressure and IAP) has also been suggested as an indirect parameter of visceral perfusion and a better survival predictor than IAP [22].

Early initiation of conservative measures has been recommended to optimize abdominal perfusion pressure, and potentially prevent surgical indication and associated morbidity and mortality. Medical management involves evacuation of abdominal contents (for example, insertion of nasogastric and rectal tubes, use of prokinetic agents, paracentesis, percutaneous drainage of space-occupying lesions); improvement of abdominal compliance (for example, sedation, analgesia, neuromuscular blockade, head of bed elevation <30 degrees); optimization of systemic perfusion (for example, judicious use of intravenous fluid and vasoactive medications); and IAP monitoring at least every 4 hours [4, 23]. Refractory cases evolving with IAP >20 mmHg with progressive organ failure should be promptly considered for surgical decompression, since delayed surgical indication has been associated with increased mortality [24, 25].

In our reported case of late post-hepatectomy hemorrhage, our patient’s IAP was 21 mmHg and it was associated with renal and respiratory failure. This clearly fulfilled the diagnostic criteria for overt ACS. Low abdominal compliance was suspected on physical examination as he had a very tense abdominal cavity with little distension. This was an expected finding in this 68-year-old overweight man with an edematous and retracted abdominal wall (due to recent and multiple past abdominal surgeries). Furthermore, the total crystalloid volume resuscitation was 4 L in the initial 24 hours, bordering reported cut-off values for adverse outcomes. Even in the presence of these contributing factors, explaining the abdominal mechanics with less than 2 L of hemoperitoneum requires reconsideration of the hydraulic principle in this case.

The hydraulic model conceives the abdomen as a muscular sac of fluid-like viscera, in which pressure is transmitted homogenously throughout. This concept assumes intra-abdominal organ-shape stability and its applicability has been proven in studies with anesthetized dogs in the absence of intra-abdominal organ deformation [26, 27]. Tissue deformation is associated with shearing forces and spatially diverse pressure gradients, which can be attenuated by intraperitoneal infusion of saline [28]. Shearing forces should be minimal in a typical patient with ACS, a fully sedated edematous patient with some degree of ascites, but not in cases of space-occupying lesions in the retroperitoneum (retroperitoneal hematoma, renal allograft) or within a solid organ (hepatic hematoma). In these cases, a decreasing pressure gradient moving away from the lesion is caused by tissue deformation, and it blunts the accuracy of indirect measures of compartment pressure. We suspect that the local supramesocolic pressures were even higher however; an impression based on the dramatic tension noted when the compartment was entered. Thus, just as direct physical pressure can be transmitted between contiguous body compartments – a phenomenon now recognized as poly-compartment syndrome – pressure can also be transmitted within compartments, as intra-compartment syndrome. For pressure transmission between the thorax and abdomen this hydraulic transmission index has been reported as being both variable and calculable from other measurements, but can be estimated using the average abdomino-thoracic index of transmission (50 % of the measured IAP) [29–31]. Potentially, ratios between intragastric and intravesical pressures might be obtainable to study this phenomenon further.

**Fig. 4:**
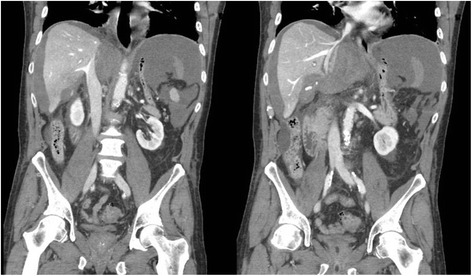
Coronal images of the computed tomography showing focal compression of the retrohepatic inferior vena cava

This case describes an unusual presentation of late post-hepatectomy hemorrhage with subsequent ACS. More interestingly, it illustrates the importance of abdominal adhesions on the fluid mechanics of the abdominal compartment. Extensive adhesions restricted intra-abdominal fluid flow and pressure transmission, thus generating tissue deformation and a non-gravitational pressure gradient. This situation was depicted on cross-sectional imaging as accumulation of fluid limited to his supramesocolic region in association with a focal compression of his retrohepatic inferior vena cava (Fig. 4). Intravesical pressure therefore underrepresented the true pressure exerted by his hemoperitoneum locally on his inferior vena cava and diaphragm, and resultant renal and respiratory dysfunctions. Intraoperative findings of highly pressurized intraperitoneal blood restricted to his supramesocolic region, and prompt improvement of respiratory pressures after drainage further support the non-hydraulic behavior of his abdominal cavity in this case of ACS.

## Conclusions

IAP and radiologic findings of acute abdominal fluid accumulation should be carefully interpreted in patients with suspected or confirmed extensive abdominal adhesions. In such cases an abdominal intra-compartment syndrome might be occurring. Once non-hydrostatic IAP gradients arise, the clinical value of indirect techniques of pressure measurement is unknown and thus a high index of suspicion is paramount in the timely diagnosis of ACS. Abdominal compliance determines intra-abdominal volume–pressure relationship. Low abdominal compliance is associated with accelerated pressure increase in response to an intra-abdominal volume expansion, and it may be suspected based on risk factors and physical examination. Aggressive fluid resuscitation has been associated with worse outcomes, and judicious fluid administration is recommended.

## Abbreviations

ACS: Abdominal compartment syndrome
HCO_3_: Bicarbonate
IAH: Intra-abdominal hypertension
IAP: Intra-abdominal pressure
pCO_2_: Partial pressure of carbon dioxide
pO_2_: Partial pressure of oxygen
